# C-reactive protein in critically ill cancer patients with sepsis: influence of neutropenia

**DOI:** 10.1186/cc10242

**Published:** 2011-05-19

**Authors:** Pedro Póvoa, Vicente Ces Souza-Dantas, Márcio Soares, Jorge IF Salluh

**Affiliations:** 1 Polyvalent Intensive Care Unit, Hospital de São Francisco Xavier, Centro Hospitalar de Lisboa Ocidental, Estrada do Forte do Alto do Duque, 1449-005 Lisboa, Portugal; 2 CEDOC, Faculty of Medical Sciences, New University of Lisbon, Campo dos Mártires da Pátria, 130, 1169-056 Lisboa, Portugal; 3 Postgraduation Program, Instituto Nacional de Câncer - INCA; Centro de Tratamento Intensivo - 10° Andar, Praça Cruz Vermelha, 23, Rio de Janeiro - RJ, CEP: 20230-130, Brazil; 4 D'Or Institute for Research and Education, Rua Diniz Cordeiro, 30, Botafogo, Rio de Janeiro-RJ, Brazil

## Abstract

**Introduction:**

Several biomarkers have been studied in febrile neutropenia. Our aim was to assess C-reactive protein (CRP) concentration in septic critically ill cancer patients and to compare those with and without neutropenia.

**Methods:**

A secondary analysis of a matched case-control study conducted at an oncologic medical-surgical intensive care unit (ICU) was performed, segregating patients with severe sepsis/septic shock. The impact of neutropenia on CRP concentrations at admission and during the first week of ICU stay was assessed.

**Results:**

A total of 154 critically ill septic cancer patients, 86 with neutropenia and 68 without, were included in the present study. At ICU admission, the CRP concentration of neutropenic patients was significantly higher than in non-neutropenic patients, 25.9 ± 11.2 mg/dL vs. 19.7 ± 11.4 mg/dL (*P *= 0.009). Among neutropenic patients, CRP concentrations at ICU admission were not influenced by the severity of neutropenia (< 100/mm^3 ^vs. ≥ 100/mm^3 ^neutrophils), 25.1 ± 11.6 mg/dL vs. 26.9 ± 10.9 mg/dL (*P *= 0.527). Time dependent analysis of CRP from Day 1 to Day 7 of antibiotic therapy showed an almost parallel decrease in both groups (*P *= 0.335), though CRP of neutropenic patients was, on average, always higher in comparison to that of non-neutropenic patients.

**Conclusions:**

In septic critically ill cancer patients CRP concentrations are more elevated in those with neutropenia. However, the CRP course seems to be independent from the presence or absence of neutropenia.

## Introduction

The frequency of cancer patients requiring intensive care has increased dramatically over the last decades [1]. Frequently, in these patients, combined mechanisms of immunosuppression coexist resulting in an increased risk for sepsis. Infection is a feared and life-threatening complication in cancer patients, in particular if neutropenia is present, that is frequently related to cancer treatments, either radiation or chemotherapy [2]. Besides, the diagnosis of infection is often difficult since the early symptoms and signs of sepsis, namely the systemic inflammatory response syndrome (SIRS), can be influenced by a number of non-infectious factors present in hemato-oncological patients [3].

Fever is probably the most commonly used clinical sign [4]. However, fever is not specific of infection since some tumours as well as chemotherapy are characteristically associated with fever, and in addition steroids, used in some cancer treatments, are very effective antipyretics [5]. The white cell count (WCC) is also not very useful since it can be markedly influenced by the cancer itself as well as by the exposure to corticosteroids and chemotherapy.

As a result early manifestations of infection are often misleading, in particular in the presence of neutropenia. Moreover, untreated infections in cancer patients can rapidly lead to a fatal outcome but, treating non-infectious causes with antimicrobials is ineffective, delays the correct treatment of the underlying disease and also increases costs, toxicity and the risk for the development of bacterial resistance represent a serious complication [6].

As a result of these limitations of the current clinical and laboratory parameters in the prompt diagnosis of infection, clinical research tried to identify mediators of the inflammatory cascade [7], that might help in that diagnosis. Several potential biomarkers of infection have been assessed in the evaluation of febrile neutropenic patients, like interleukin (IL)-6, IL-8, serum amyloid A, C-reactive protein (CRP), procalcitonin [8,9], with diverse results.

Almost all studies assessed the diagnostic and/or prognostic performance of different biomarkers of infection in septic cancer patients, namely with febrile neutropenia. However, non-neutropenic cancer patients with sepsis are usually excluded from these studies. In the present study, our aim was to assess in septic cancer patients the concentrations of a widely used biomarker of infection, CRP, comparing the baseline concentrations and response to antibiotic therapy in those with and without neutropenia.

## Materials and methods

### Design and setting

The present study is a secondary analysis of a matched case-control study performed in the ICU of Instituto Nacional de Câncer (INCa), Rio de Janeiro, Brazil. Details of the study design, definitions and data collection are provided elsewhere [10]. Briefly, during the study period (January 2003 to July 2007), every adult cancer patient (≥ 18 yrs) that required ICU admission due to life-threatening complications was consecutively enrolled. Patients in complete remission of more than 5 yrs, those with an ICU stay less than 24 hrs and readmissions were not considered. The ICU is a 10-bed medical-surgical unit specialized in the care of patients with cancer [11,12], with the exception of bone marrow transplant patients.

This study was supported by institutional funds and did not interfere with clinical decisions related with patient care. The Local Ethics Committee approved the study (N° 10/2003) and the need for informed consent was waived.

### Definitions, selection of participants and data collection

Infection was defined as the presence of a pathogenic microorganism in a sterile milieu (such as blood or cerebrospinal fluid) and/or clinically suspected infection that justified the administration of antibiotics [13,14]. Sepsis severity was classified according to the consensus conference definitions [15].

Neutropenia was defined as a neutrophil count below 500/mm^3 ^[2]. Neutropenia was further classified as chemotherapy related or unrelated. During the study period, from a prospective cohort of 1,332 consecutive cancer patients, 94 patients with neutropenia and well-matched controls without neutropenia, in a 1:1 ratio, were compared [10]. For the present study, cancer patients with sepsis were segregated, 86 neutropenic and 68 non-neutropenic. Empiric antibiotic therapy was started in all septic cancer patients upon ICU admission according to to local guidelines and in accordance with the Infectious Diseases Society of America guidelines [2]. The prescription was not delayed by the collection of appropriate samples for microbiological cultures. At least two blood cultures were performed from independent venipunctures in each newly admitted patient. Additional samples for microbiological cultures were collected according to the suspected primary focus of infection.

Demographic, clinical and laboratory data were collected using standardized case report forms during the first day of ICU stay including main diagnosis for admission, the Simplified Acute Physiology Score (SAPS) II [16], the Sequential Organ Failure Assessment (SOFA) score [17], comorbidities, and cancer- and treatment-related data. For the purpose of the present study, individual organ failures were diagnosed in case of a SOFA score ≥ 2 points in each domain [14]. In addition, patients receiving dialysis in the context of acute kidney injury and invasive mechanical ventilation (MV) on the first day of ICU were considered as having renal and respiratory failures regardless the SOFA score, respectively. The ICU and hospital mortality rates were also assessed.

Blood samples were obtained via an arterial line on admission and, subsequently, every morning at 07:00 hrs. Measurement of CRP was performed by means of an immunoturbidimetric method using a commercially available kit (Tina-quant CRP; Roche Diagnostics, Mannheim, Germany). The precision of the assay measured by means of the intra- and inter-assay coefficient of variation was < 7%, the sensitivity 0.1 mg/dL and the detection limit 0.3 mg/dL. C-reactive protein was measured during the first week of ICU stay at Day 1 (D1), D3, D5 and D7.

CRP concentrations at ICU admission and during the first week of sepsis course were analysed, comparing neutropenic with non-neutropenic septic critically ill cancer patients.

### Data processing and statistical analysis

Data entry was performed by a single investigator (MS) and consistency was assessed with a rechecking procedure of a 10% random sample of patients. Data were screened in detail by three investigators (MS, JIFS, VCSD) for missing information, implausible and outlying values.

Continuous variables were reported as mean ± standard deviation or median (25% to 75% interquartile range, IQR) according to data distribution. Comparisons between groups were performed using the parametric unpaired and paired t-test, or the nonparametric Mann-Whitney U test and Wilcoxon signed-rank test for continuous variables according to data distribution. The Chi-square test was used to carry out comparisons between categorical variables. Correlations were calculated by the Spearman's rank correlation. Time-dependent analysis of CRP was performed via General Linear Model univariate repeated-measures analysis using a split-plot design approach.

In all cases, statistical significance was defined as a two-tailed test with an alpha of 0.05. All statistical calculations were preformed using the PASW v. 18.0 for MAC (SPSS, Chicago, IL, USA).

## Results

### Characteristics of the study population

A total of 154 critically ill septic cancer patients were included in the present study, 86 with neutropenia, that represents all neutropenic septic cancer patients admitted in the ICU during the study period, and the remainder without neutropenia (*N *= 68). The patients' main characteristics are depicted in Table 1. The sources of ICU admission were the operating room (10.4%), emergency department (16.9%) and wards (72.7%) (*P *= 0.238, comparing neutropenic vs. non-neutropenic patients). There were 105 (68.2%) patients with hematological malignancies and 49 (31.8%) with solid tumors (*P *= 0.569). The most frequent underlying malignancies were lymphomas (*N *= 59, 38.3%), leukemias (*N *= 32, 20.8%), gastrointestinal (*N *= 13, 8.4%), multiple myeloma (*N *= 9, 5.8%), urogenital (*N *= 8, 5.2%) and others (*N *= 33, 21.4%). Previous anticancer treatments included surgery for tumor resections (3.9%), chemotherapy (72.7%) and radiation therapy (23.4%). Comorbidities were indentified in 129 (83.8%) patients and the most frequent were immunosuppression (40.3%), arterial hypertension (20.4%), acquired immunodeficiency syndrome (8.4%), diabetes mellitus (6.5%) and chronic obstructive pulmonary disease (6.5%).

**Table 1:** Baseline patients' characteristics and comparison between neutropenic and non-neutropenic patients

	All Patients	Neutropenic	Non neutropenic	*P*-value
N	154	86	68	
Age (yrs)	48.5 ± 18.1	47.0 ± 17.8	50.4 ± 18.4	0.248
Gender (M/F)	94/60	54/32	40/28	0.622
Type of cancer				0.569
Solid	49	29	20	
Hematologic	105	57	48	
Previous radiotherapy	36	21	15	0.731
Previous Chemotherapy	112	72	40	0.001
Previous surgery	6	1	5	0.049
Non-invasive Ventilation	15	14	1	0.002
Invasive mechanical Ventilation	135	74	61	0.493
Vasopressors	112	62	50	0.842
Type of infection				0.007
Pneumonia	63	28	35	
Peritonitis	15	7	8	
Urinary	3	0	3	
Blood stream infections	8	4	4	
Skin/Soft tissue infections	7	4	3	
CNS infections	1	0	1	
Other infections	57	43	14	
SAPS II (points)	62.2 ± 16.8	62.2 ± 16.7	62.5 ± 16.8	0.827
SOFA (Day 1) (points)	11.4 ± 3.9	11.6 ± 4.1	11.2 ± 4.1	0.591
Sepsis severity				0.899
Sepsis	10 (6.5%)	6 (7%)	4 (5.9%)	
Severe sepsis	29 (18.8%)	17 (19.8%)	12 (17.6%)	
Septic shock	111 (74.7%)	63 (73.3%)	52(76.5%)	
Total white cell count (/mm^3^)	1,400 (14,636)	352 (909)	22,100 (35,900)	< 0.001
Temperature (°C)	37.0 ± 1.5	37.2 ± 1.5	36.8 ± 1.5	0.119
CRP (Day 1) (mg/dL)	23.6 ± 11.6	25.9 ± 11.2	19.7 ± 11.4	0.009
Duration of mechanical ventilation (days)	6.0 (9.0)	6.0 (8.0)	6.0 (9.0)	0.616
ICU length of stay (days)	7.0 (10.3)	7.0 (12.0)	8.0 (10.0)	0.699
Hospital length of stay (days)	18.5 (23.6)	20.5 (25.0)	16.5 (21.0)	0.111
ICU mortality	111 (72.1%)	60 (69.8%)	51 (75.0%)	0.472
Hospital mortality	122 (79.2%)	65 (75.6%)	57 (83.8%)	0.211

Values expressed as N (%), mean ± standard deviation or median (interquartile range] according to type of data and data distribution; abbreviations: CNS, central nervous system; CRP, C-reactive protein; ICU, intensive care unit; SAPS II, Simplified Acute Physiology Score II; SOFA, Sequential Organ Failure Assessment score

The length of ICU and hospital stay were (median (IQR)) 7.0 (10.3) days and 18.5 (23.6) days, respectively, without significant differences between neutropenic and non-neutropenic patients (*P *= 0.699 and *P *= 0.111, respectively). The overall ICU and hospital mortality rates were 72.1% and 79.2%, respectively, without significant differences between study groups (*P *= 0.472 and *P *= 0.211, respectively).

Most of the patients were admitted in the ICU in severe sepsis/septic shock (93.5%) as well as with a severe degree of organ failure/dysfunction (SOFA at D1, 11.4 ± 3.9 points).

Almost two-thirds of the infections were microbiologically proven infections (65.1%). As expected, the most frequent sites of infection were the lungs, abdomen and bloodstream infection. Gram-negative bacteria were responsible for 72.2% of the infection episodes and 26 (36.6%) patients had polymicrobial (more than one infectious agent) infections.

### Impact of neutropenia on temperature and C-reactive protein

**Figure 1:**
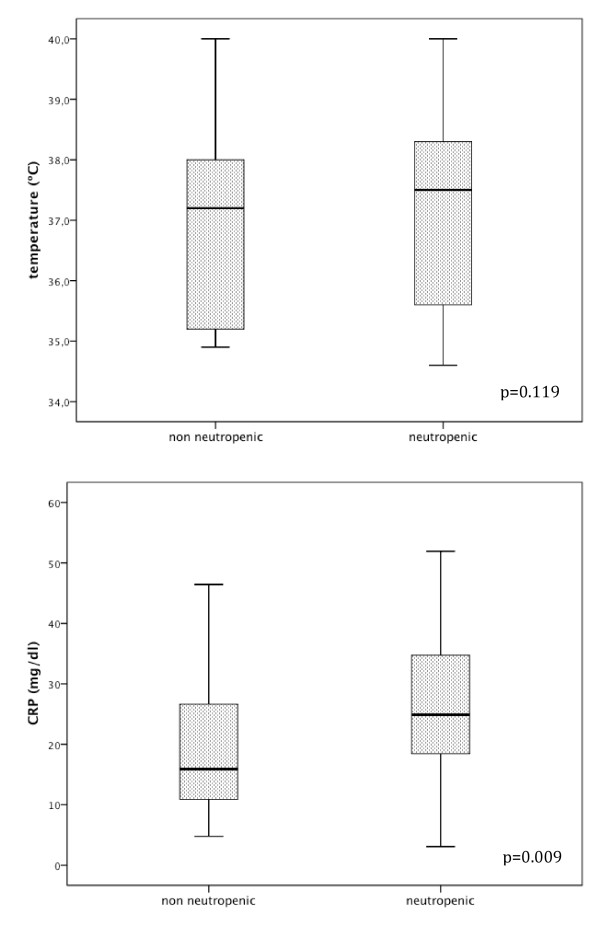
**Temperature and C-reactive protein of neutropenic and non-neutropenic septic cancer patients at ICU admission**. Comparison of temperature (°C) and C-reactive protein concentrations (mg/dL) at ICU admission between neutropenic and non-neutropenic septic critically ill cancer patients (*P *= 0.119 and *P *= 0.009, respectively).

At ICU admission, temperature in septic critically ill cancer patients was not significantly different in those presenting neutropenia in comparison with non-neutropenic patients (37.2 ± 1.5°C vs. 36.8 ± 1.5°C, respectively, *P *= 0.119) (Figure 1). Concerning CRP (Figure 1), we found that neutropenic septic cancer patients showed a significantly higher concentration, 25.9 ± 11.2 mg/dL, in comparison with CRP concentration from non-neutropenic patients, 19.7 ± 11.4 mg/dL (*P *= 0.009). Additionally, among neutropenic patients CRP concentrations at ICU admission were not influenced by the severity of neutropenia (< 100/mm^3 ^vs. ≥ 100/mm^3 ^neutrophils), 25.1 ± 11.6 mg/dL vs. 26.9 ± 10.9 mg/dL, respectively (*P *= 0.527).

We also assessed the correlation between WCC and CRP concentration. We found a poor, whilst significant, correlation between these two variables (r*_s _*= -0.252, *P *= 0.012).

### C-reactive protein course in neutropenic and non-neutropenic patients

**Figure 2:**
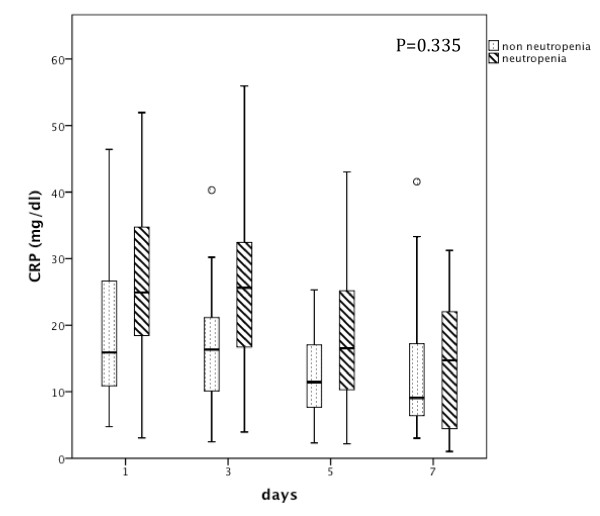
**C-reactive protein course of neutropenic and non-neutropenic septic critically ill cancer patients**. Time course of CRP concentrations (mg/dL) for neutropenic and non-neutropenic septic critically ill cancer patients during the first week of antibiotic therapy (*P *= 0.335).

Time dependent analysis of CRP (Figure 2) from D1 to D7 of antibiotic therapy showed an almost parallel course in both groups (*P *= 0.335), with almost no change from D1 to D3, followed by a significant decrease from D3 onwards; though the CRP concentration of neutropenic patients was, on average, higher in comparison to that of non-neutropenic patients. From D1 to D7, CRP concentration of neutropenic and non-neutropenic patients decreased from 25.9 ± 11.2 mg/dL and 19.7 ± 11.4 mg/dL at D1 to 14.1 ± 9.1 mg/dL and 13.1 ± 10.8 mg/dL at D7 (*P *< 0.001 and *P *= 0.009, respectively).

## Discussion

We found among septic critically ill cancer patients a marked increase in CRP concentrations irrespective of the WCC, at ICU admission. Even though CRP concentrations in neutropenic patients were significantly higher, we found a poor correlation between WCC and CRP concentrations. Finally, our findings demonstrate that the course of CRP during the first week of antibiotic therapy was similar in neutropenic and non-neutropenic septic critically ill cancer patients.

Since inadequately treated infections can be rapidly fatal in neutropenic cancer patients, a great deal of clinical research on biomarkers has been published [8,9]. Several biomarkers, such as IL-6, IL-8, CRP, brain natriuretic peptides, procalcitonin, neopterin, have been evaluated in patients with febrile neutropenia to assess their performance in the diagnosis of infection [18-24], in the identification of the underlying agents [18-20,22,24], in the characterization of sepsis severity and outcome prediction [21,23-27]. However, information on biomarkers comparing neutropenic and non-neutropenic cancer patients are currently limited [28].

Among septic non-cancer patients there is substantial controversy concerning the potential effects of immunosuppression, in particular of corticosteroids, on CRP concentration, decreasing acute phase response independently of the treatment of infection [29-33].

In the present study, we clearly demonstrate that CRP, a major acute phase reactant protein, increases markedly in profoundly immunosuppressed cancer patients with sepsis. In other words, the acute phase reaction seems to remain unaffected by either chemotherapy or radiotherapy. Moreover, we found that septic neutropenic cancer patients had significantly higher CRP concentrations in comparison with non-neutropenic patients at ICU admission. Neutropenia reflects a profound state of immunosuppression representing a markedly increase susceptibility to infections [4]. In addition, neutropenic patients present an increased risk to acquire infections caused not only by "common" bacteria, but also by opportunistic agents, like virus and fungi, secondary to a decrease cellular and humoral immunity [4]. In addition, the size of the inoculum necessary to produce an infection is reduced in neutropenic patients. In this context, we could hypothesize that microbiological agents would invade and proliferate easily in neutropenic patients, reaching a higher microbiological burden and also leading to a larger inflammatory response, reflected by a higher CRP concentration [34-36].

Consequently, our findings pointed to the clinical usefulness of CRP in critically ill septic cancer patients irrespective of the presence or absence of neutropenia, as well as, the degree of neutropenia.

Interestingly, other commonly used biomarkers in non-cancer patients, such as PCT, should be used with some reserve in neutropenia. The origin of PCT in the inflammatory response is not yet fully understood [37]. Moreover, it has been shown that in septic cancer patients with leukopenia PCT concentrations were lower when compared with patients without leukopenia [28]. Consequently, it is possible to observe PCT values < 0.5 ng/ml in infected febrile neutropenic patients [9].

Besides, we recognize that the present study has some limitations. First, our study was an observational single centre study. Second, clinical and laboratory data assessing the recovery phase of neutropenia and factors that could have influenced the CRP course were not routinely collected. Third, since we only assessed CRP course during the first week of antibiotic therapy we cannot draw any conclusion concerning CRP course beyond D7. However, our study has also several important strengths. To date, this is the first study comparing CRP concentrations in septic cancer patients with and without neutropenia, and with a large cohort of septic neutropenic patients.

## Conclusions

In conclusion, the results of this study provide valuable information concerning the CRP biology and time-course in septic critically ill cancer patients. It was clear from our results that septic cancer patients express a full blown acute phase response with marked CRP elevations, and that this was particularly significant in the presence of neutropenia. Finally, CRP course was not influenced by the presence or absence of neutropenia. As a result, CRP could be a clinically useful bedside biomarker of infection in cancer patients irrespective of the WCC and the degree of immunosuppression.

## Key messages

• In the present study we showed that septic cancer patients express a full blown acute phase response with marked CRP elevations, and that this was particularly significant in the presence of neutropenia.

• The CRP course during the first week of antibiotic therapy was not influenced by the presence or absence of neutropenia.

• CRP could be a useful biomarker of infection in cancer patients irrespective of the WCC and the degree of immunosuppression.

## Abbreviations

CRP: C-reactive protein; ICU: intensive care unit; IL: interleukin; IQR: interquartile range; MV: mechanical ventilation; SAPS II: Simplified Acute Physiology Score (SAPS) II; SIRS: systemic inflammatory response syndrome; SOFA: Sequential Organ Failure Assessment; WCC: white cell count.

## Competing interests

PP has received honoraria and served as advisor of Astra Zeneca, Ely-Lilly, Gilead, Janssen-Cilag, Merck Sharp & Dohme, Novartis and Pfizer and received an unrestricted research grant from Brahms and Virogates. VCSD, MS and JIFS have no competing interests to declare.

## Authors' contributions

PP, VCSD, MS and JIFS contributed to the study conception and design, carried out and participated in data analysis and drafted the manuscript. VCSD, MS and JIFS participated in acquisition of data. All authors read and approved the final version of the manuscript.

## Authors' information

PP is coordinator of the Polyvalent Intensive Care Unit and president of the Antibiotic Commission of São Francisco Xavier Hospital. PP is Professor of Medicine of the Faculty of Medical Sciences from the New University of Lisbon, Portugal. VCSD is assistant physician of the ICU of the Instituto Nacional de Câncer, Rio de Janeiro, Brazil. MS and JIFS are associate investigators of D'Or Institute for Research and Education.
